# Low hepatitis C antibody screening rates among an insured population of Tennessean Baby Boomers

**DOI:** 10.1371/journal.pone.0188624

**Published:** 2017-11-30

**Authors:** James G. Carlucci, Syeda A. Farooq, Lindsey Sizemore, Michael Rickles, Brandon Cosley, Leigh McCormack, Carolyn Wester

**Affiliations:** 1 Vanderbilt University Medical Center, Nashville, Tennessee, United States of America; 2 Tennessee Department of Health, Nashville, Tennessee, United States of America; 3 Collaborating Health Insurer, Chattanooga, Tennessee, United States of America; Centers for Disease Control and Prevention, UNITED STATES

## Abstract

**Introduction:**

Chronic Hepatitis C Virus (HCV) infection is common and can cause liver disease and death. Persons born from 1945 through 1965 ("Baby Boomers") have relatively high prevalence of chronic HCV infection, prompting recommendations that all Baby Boomers be screened for HCV. If chronic HCV is confirmed, evaluation for antiviral treatment should be performed. Direct-acting antivirals can cure more than 90% of people with chronic HCV. This sequence of services can be referred to as the HCV "cascade of cure” (CoC). The Tennessee (TN) Department of Health (TDH) and a health insurer with presence in TN aimed to determine the proportion of Baby Boomers who access HCV screening services and appropriately navigate the HCV CoC in TN.

**Methods:**

TDH surveillance data and insurance claim records were queried to identify the cohort of Baby Boomers eligible for HCV testing. Billing codes and pharmacy records from 2013 through 2015 were used to determine whether HCV screening and other HCV-related services were provided. The proportion of individuals accessing HCV screening and other steps along the HCV CoC was determined. Multivariable analyses were performed to identify factors associated with HCV screening and treatment.

**Results:**

Among 501,388 insured Tennessean Baby Boomers, 7% were screened for HCV. Of the 40,019 who received any HCV-related service, 86% were screened with an HCV antibody test, 20% had a confirmatory HCV PCR, 9% were evaluated for treatment, and 4% were prescribed antivirals. Hispanics were more likely to be screened and treated for HCV than non-Hispanic whites. HCV screening was more likely to occur in the Nashville-Davidson region than in other regions of TN, but there were regional variations in HCV treatment.

**Conclusions:**

Many insured Tennessean Baby Boomers do not access HCV screening services, despite national recommendations. Demographic and regional differences in uptake along the HCV CoC should inform public health interventions aimed at mitigating the effects of chronic HCV.

## Introduction

Hepatitis C virus (HCV) infection is the most common blood-borne disease in the United States (US) with approximately 3.5 million people living with chronic HCV [1]. Tennessee (TN), along with three other states in Central Appalachia, had more than a threefold increase in acute HCV infections from 2006 to 2012 among individuals aged 30 years or younger [2], nearly double the national rate [3]. Once infected with HCV, 75–85% of acutely infected persons develop chronic disease, potentially resulting in liver damage, cancer, and eventually death [4]. However, with proper diagnosis and treatment, a person can be cured of HCV.

“Baby Boomers" have been identified as a group with high prevalence of chronic HCV, accounting for nearly three-fourths of all chronic HCV infections in the US [5]. Since 2013 the US Centers for Disease Control and Prevention (CDC) and the US Preventative Services Task Force (USPSTF) have recommended that all people born from 1945 through 1965 be tested for HCV at least once in their lifetime [5, 6]. Despite this recommendation, previous studies have shown that only about half of those chronically infected with HCV are diagnosed and aware of their status, and fewer than 10% complete the cascade of diagnostic and treatment services that can result in cure [1].

If a screening HCV antibody test is positive, confirmatory molecular PCR testing should be performed to determine if the patient is currently infected with HCV. Once current HCV infection is confirmed, further staging and evaluation for treatment eligibility should be performed, and if indicated a person can be treated with antiviral medications. New direct-acting antivirals (DAAs) have revolutionized HCV treatment and can cure more than 90% of people with chronic HCV infection [7]. This sequence of diagnostic and treatment services can be referred to as the HCV "cascade of cure” (CoC).

The TN Department of Health (TDH) systematically collects statewide HCV test results for surveillance purposes. Reporting of acute HCV has been mandatory since the 1990s. Laboratory-based reporting of chronic HCV was mandated beginning January 1, 2017.

A large health insurer with a presence in TN collects data associated with healthcare services incurred by their clients. These data include diagnoses, procedures, place of service, costs, and provider-related information and are captured purely for reimbursement purposes. However, there is the potential for these data to support public health surveillance efforts, such as the HCV CoC. These data include HCV-related lab tests, imaging, sub-specialist visits, and prescriptions for anti-HCV drugs, including DAAs, when available. Intermittent and varying contractual obligations may limit data collection, standardization, and availability.

In TN, the proportion of Baby Boomers who access HCV screening services and factors associated with accessing HCV-related health services have not been well studied. The objective of this study was to address these knowledge gaps in order to inform strategies for providing more targeted support to populations at risk for and affected by HCV in TN.

## Methods

### Study design and data sources

We performed a retrospective observational cohort study using information from medical and pharmacy claims and TDH databases. Medical claim records were queried to identify a cohort of Tennesseans at-risk for chronic HCV infection. Persons who were born from 1945 through 1965, had at least one clinical or laboratory encounter from January 1, 2013 through December 31, 2015, and were continuously enrolled with the insurer for at least 6 months from the first clinical encounter during the study period were included in the cohort. TDH surveillance data were used to identify and exclude individuals who were diagnosed with acute and/or chronic HCV prior to 2013.

International Classification of Disease (ICD) codes are used by providers to document a condition, but they are not used or accepted by third-parties for billing. As such, the collaborating insurer only had access to and provided data including billing codes (Current Procedural Terminology [CPT] and Logical Observation Identifiers Names and Codes [LOINC]). These billing codes and pharmacy prescription claims were used to determine whether HCV-related services were provided to individuals in the cohort (Table 1). In addition, identification of provider subspecialties for specific visits was based on contractual assignments for a given provider’s primary contracted specialty. Claims data were queried based on the presence of specific codes used to identify HCV- and non-HCV-related primary diagnoses and/or primary procedures falling within the study timeframe.

**Table 1:** Diagnostic/billing codes and medications queried to determine whether HCV-related services were provided.

Code type and number	Explanation
**CPT**	
74150	Abdominal CT scan
74181	MRI abdomen with contrast
74182	MRI abdomen without contrast
74183	MRI abdomen with and without contrast
76700	Abdominal ultrasound
81599	Multianalyte assay with algorithmic analysis
86803	HCV antibody
86804	HCV antibody, confirmatory test (with reflex)
87520	HCV, direct probe technique
87521	HCV, amplified probe technique
87522	HCV, quantification
87902	HCV genotype test
91200	Transient elastography
G0472	HCV antibody screening, for an individual at high risk
**LOINC**	
11011–4	HCV RNA
11259–9	HCV RNA in Serum/Plasma by probe and target amplification
13955–0	HCV antibody
1742–6	Alanine aminotransferase*
1835–8	Alpha-2-macroglobulin*
1869–7	Apolipoprotein A-I*
1975–2	Total bilirubin*
32286–7	HCV genotype
2324–2	Gamma-Glutamyl Transferase*
38180–6	HCV RNA
4542–7	Haptoglobin*
48159–8	HCV antibody
48792–6	Necroinflammatory activity score*
48793–4	Necroinflammatory activity grade*
48794–2	Fibrosis stage*
48795–9	Fibrosis score*
**Medications**	
**Class**	**Generic name**
Direct-acting antivirals**	Boceprevir
	Daclatasvir
	Dasabuvir
	Elbasavir
	Grazoprevir
	Ledipasvir
	Ombitasvir
	Ritonavir
	Simeprevir
	Sofosbuvir
	Telaprevir
**Interferons**	Peginterferon alfa-2a
	Peginterferon alfa-2b
	Interferon alfa-2b
	Interferon alfacon-1
**Ribavirin**	

Definitions: computed tomography (CT), magnetic resonance imaging (MRI), ribonucleic acid (RNA).

* Components of multianalyte assay with algorithmic analysis; not independently considered to be HCV-related codes.

** Fixed-dose combinations of direct-acting antivirals were also captured by our search.

### Data merger

After the medical and pharmacy claims database was queried and the cohort constructed, the data were securely transmitted to partners at TDH. There were 622,349 records received from the insurer that met the inclusion criteria, and after de-duplication 503,204 unique persons remained in the claims set (if someone changed jobs and enrolled with the insurer through a new plan, the individual may be listed twice in the original query). Using a three-step algorithm these data were linked to and merged with 67,183 records from the National Electronic Disease Surveillance System (NEDSS) Based System (NBS), representing unique HCV conditions prior to and including 2016. Test result information for 6,059 acute and/or chronic HCV- positive clients matched between the databases and was added to the main claims file. After this merger, names were replaced with unique identification numbers, addresses were replaced by public health department regions [8], dates of birth were converted to age in years (as of December 31, 2016), and all other potential personal identifiers were removed.

### Definitions

Those who met inclusion criteria and had not been diagnosed with HCV prior to the beginning of the study period were considered *eligible for HCV screening*. Those *screened* for HCV were those who had an HCV antibody test performed or who had an HCV antibody test result reported. Those with *confirmatory testing* were those who had an HCV qualitative or quantitative PCR test performed or an HCV PCR result reported (individuals who had an HCV PCR test did not necessarily have a screening HCV antibody test performed during the study period). Those *evaluated for treatment* were those who: had an HCV genotype; had a multianalyte assay; were evaluated by a specialist (Gastroenterologist/Hepatologist or Infectious Diseases) and had a positive HCV PCR test; and/or had abdominal/liver imaging and a positive HCV PCR test. If a person had been prescribed any anti-HCV medication, they were considered to have been *treated*.

### Statistical analyses

Descriptive statistics were used to summarize the characteristics of the cohort. Continuous variables are reported as median and interquartile range (IQR). Categorical variables are reported as percentages. Proportions of individuals completing each step of the HCV CoC were calculated among the entire population eligible for HCV screening and among those who had at least one HCV-related code or prescription. Specifically, we calculated: (a) the proportion of those who received an HCV antibody test among those eligible for screening, (b) the proportion of those who received an HCV antibody test among those who received any HCV-related service, (c) the proportion of those who received an HCV PCR among those who received any HCV-related service, (d) the proportion of these who received an evaluation for HCV treatment among those who received any HCV-related service, and (e) the proportion of those treated with an anti-HCV medication among those who received any HCV-related service. Multivariable logistic regression was used to identify factors associated with HCV screening among those eligible for screening, and factors associated with receiving treatment among those who were evaluated for treatment. Odds ratios (OR) with 95% confidence intervals (CI) are reported. Race/ethnicity data were missing for 71% of individuals, therefore census data were used to impute the missing values proportionate to the racial/ethnic distribution within each of TN’s public health regions. Microsoft Excel [2010], Statistical Analysis Software (SAS) Version 9.3 (SAS Institute, Inc., Cary, NC), and R version 3.2.3 (R Foundation for Statistical Computing, Vienna, Austria) were used for statistical analyses.

### Ethics approval

This study has been approved by Vanderbilt University (IRB# 161394) and TDH (IRB# 930766). The collaborating insurer did not require IRB approval because this research is considered a health care operation furthering population-based activities related to improving health for all Tennesseans.

## Results

**Fig 1:**
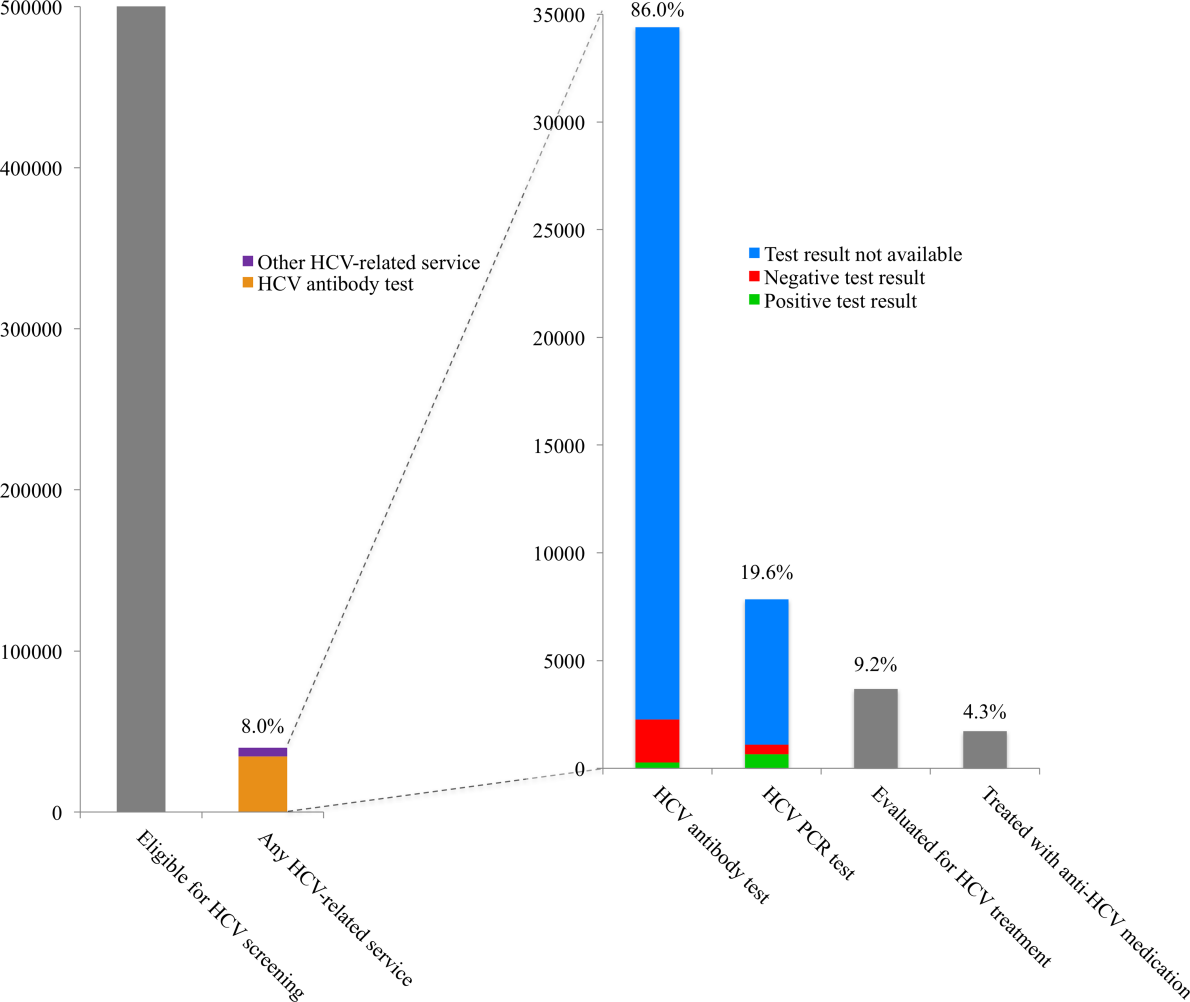
Chronic hepatitis C cascade. Frequency and proportions of insured Tennessean Baby Boomers accessing various HCV-related services, among those eligible for HCV screening and among those who received any HCV-related service.

After excluding 1,816 individuals who had been diagnosed with acute and/or chronic HCV prior to the study period (prior to 2013), there were 501,388 Tennessean Baby Boomers eligible for HCV screening. Of these, 40,019 (8%) had at least one HCV-related code, and 34,403 (7%) had an HCV antibody test performed (Fig 1). There were more women (54%) than men (46%), and the sample was predominantly non-Hispanic white (81%). Age, sex, race, and regional distributions were similar between those who were screened for HCV and those who were not screened for HCV. (Table 2)

**Table 2:** Distribution of patient characteristics, stratified by whether screening with an HCV antibody test was performed.

	Entire Population n = 501388	HCV antibody test n = 34403	No HCV antibody test n = 466985
**Age in years, median (IQR)**	59 (54–64)	58 (54–63)	59 (54–64)
**Sex, n (%)**			
Female	271027 (54.1%)	18847 (54.8%)	252180 (54.0%)
Male	229968 (45.9%)	15545 (45.2%)	214423 (45.9%)
Missing	393 (<0.1%)	11 (<0.1%)	382 (<0.1%)
Race*, n (%)			
Non-Hispanic Asian	7063 (1.4%)	484 (1.4%)	6579 (1.4%)
Non-Hispanic Black	71188 (14.2%)	5975 (17.4%)	65213 (14.0%)
Hispanic	2285 (0.5%)	416 (1.2%)	1869 (0.4%)
Non-Hispanic White	407310 (81.2%)	26981 (78.4%)	380329 (81.4%)
Non-Hispanic Multi-Race	6213 (1.2%)	54 (0.2%)	6159 (1.3%)
Other**	7329 (1.5%)	493 (1.4%)	6836 (1.5%)
**Region, n (%)**			
East Tennessee^†^	195351 (39.0%)	12167 (35.4%)	183184 (39.2%)
Middle Tennessee ^‡^	195787 (39.0%)	14932 (43.4%)	180855 (38.7%)
West Tennessee ^§^	100846 (20.1%)	6860 (20.0%)	93986 (20.1%)
Missing	9404 (1.9%)	444 (1.3%)	8960 (1.9%)

* 70.8% of race values have been imputed as described in the Methods.

** Includes American Indian and Native Hawaiian.

^**†**^ East Tennessee includes the Chattanooga-Hamilton, East, Knox, Northeast, Southeast, and Sullivan public health regions.

^**‡**^ Middle Tennessee includes the Mid-Cumberland, Nashville-Davidson, South Central, and Upper Cumberland public health regions.

^**§**^ West Tennessee includes the Jackson-Madison, Memphis-Shelby, and West public health regions.

Among the 40,019 who received any HCV-related service, 34,403 (86%) were screened for with an HCV antibody test, 7,848 (20%) had an HCV PCR confirmatory test, 3,690 (9%) were evaluated for treatment, and 1,731 (4%) were prescribed medication for HCV treatment (Fig 1). Of the 3690 people evaluated for HCV treatment, 435 (12%) received a multianalyte assay and/or elastography. Overall, relatively few test results were available. Among those who had an HCV antibody test, 1% were positive, 6% were negative, and results were not available for 93%. Among those who had an HCV PCR test, 8% were positive, 6% were negative, and results were not available for 86%. Due to missing PCR results, we were not able to determine rates of sustained virologic response, or cure (i.e., undetectable HCV RNA at least 12 weeks after completion of therapy).

**Fig 2:**
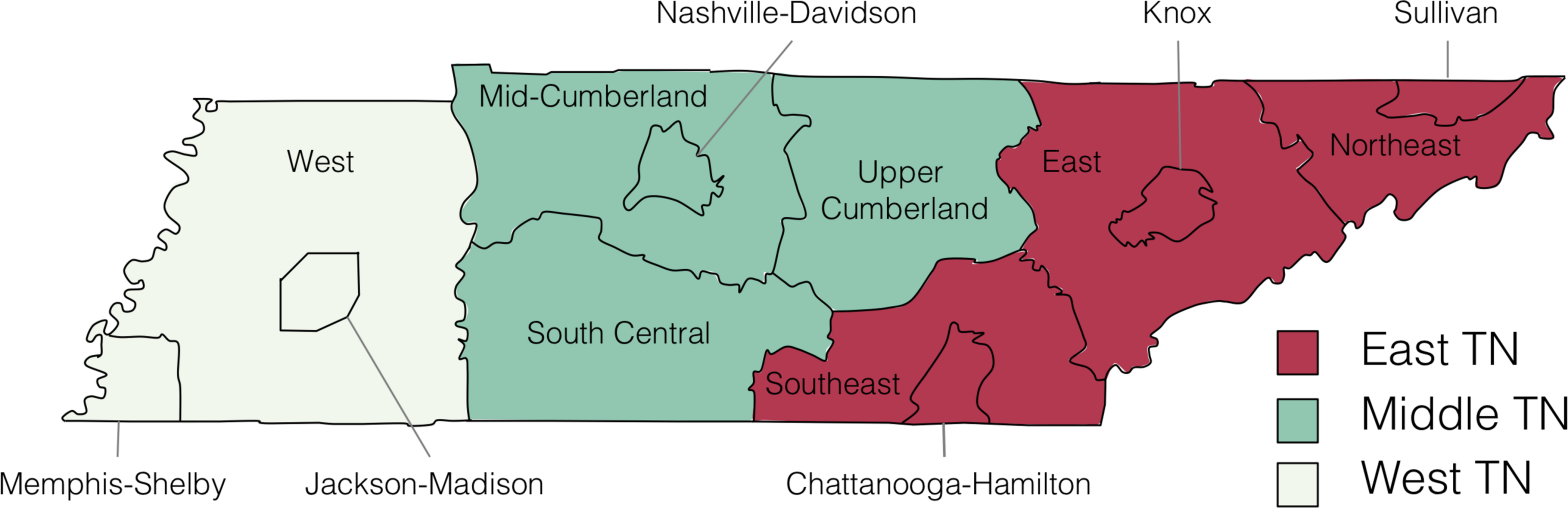
Map of Tennessee Department of Health regions. East Tennessee includes the Chattanooga-Hamilton, East, Knox, Northeast, Southeast, and Sullivan public health regions. Middle Tennessee includes the Mid-Cumberland, Nashville-Davidson, South Central, and Upper Cumberland public health regions. West Tennessee includes the Jackson-Madison, Memphis-Shelby, and West public health regions.

Among all those eligible for HCV screening, Hispanics (OR: 2.77, 95% CI: 2.49–3.09) and non-Hispanic blacks (OR: 1.10, 95% CI: 1.06–1.14) had significantly higher odds of being screened for HCV than non-Hispanic whites. Those categorized as non-Hispanic multi-race were less likely to be screened for HCV (OR: 0.11, 95% CI: 0.08–0.14). Women were more likely to be screened for HCV than men (OR: 1.03, 95% CI: 1.01–1.05). HCV screening was more likely to occur in the Nashville-Davidson region than in other regions of TN, and the odds for screening were lowest for the Jackson-Madison (OR: 0.33, 95% CI: 0.30–0.37) and Sullivan (OR: 0.38, 95% CI: 0.35–0.41) regions. (Fig 2 and Table 3)

**Table 3:** Multivariable logistic regression analyses to determine associations with (A) HCV antibody screening among the entire at-risk population, and (B) HCV treatment among those evaluated for treatment.

	A: HCV antibody screening among those eligible for screening* Odds ratio (95% confidence interval)	B: HCV treatment among those evaluated for HCV treatment** Odds ratio (95% confidence interval)
**Age (per 1 year)**	0.98 (0.98–0.98)	1.00 (0.99–1.00)
**Female (vs. male)**	1.03 (1.01–1.05)	0.95 (0.92–0.98)
**Race/Ethnicity**^†^		
Non-Hispanic White (ref)		
Non-Hispanic Asian	0.92 (0.84–1.01)	1.10 (0.92–1.31)
Non-Hispanic Black	1.10 (1.06–1.14)	1.04 (1.00–1.09)
Hispanic	2.77 (2.49–3.09)	1.28 (1.12–1.47)
Non-Hispanic Multi-Race	0.11 (0.08–0.14)	0.94 (0.59–1.50)
Other	0.93 (0.85–1.03)	1.07 (0.93–1.23)
**Region**		
Nashville–Davidson (ref)		
Chattanooga–Hamilton	0.96 (0.91–1.01)	1.09 (1.00–1.18)
East	0.56 (0.53–0.59)	1.04 (0.97–1.12)
Jackson–Madison	0.33 (0.30–0.37)	0.94 (0.83–1.05)
Knox	0.66 (0.62–0.70)	1.10 (1.02–1.18)
Mid-Cumberland	0.80 (0.77–0.84)	1.03 (0.97–1.10)
Memphis–Shelby	0.87 (0.83–0.92)	0.93 (0.87–0.99)
Northeast	0.53 (0.49–0.56)	1.13 (1.04–1.23)
South Central	0.50 (0.47–0.53)	1.10 (1.01–1.20)
Southeast	0.55 (0.52–0.60)	1.18 (1.08–1.29)
Sullivan	0.38 (0.35–0.41)	1.00 (0.89–1.11)
Upper Cumberland	0.50 (0.46–0.53)	1.06 (0.97–1.17)
West	0.52 (0.49–0.55)	1.00 (0.91–1.03)

* Multivariable logistic regression to determine the factors associated with having an antibody test performed (n = 34403) among all those eligible for screening (n = 501388).

** Multivariable logistic regression to determine the factors associated with being prescribed anti-HCV medication (n = 1262) among those who were evaluated for treatment (n = 3690).

^†^ The data for the ‘race/ethnicity’ variable have been imputed as described in Methods section of this paper.

Among those evaluated for HCV treatment, Hispanics (OR: 1.28, 95% CI: 1.12–1.47) and non-Hispanic blacks (OR: 1.04, 95% CI: 1.00–1.09) were more likely to be treated for HCV than non-Hispanic whites. Women had lower odds of receiving anti-HCV medication than men (OR: 0.95, 95% CI: 0.92–0.98). Compared to the Nashville–Davidson region, HCV treatment was less likely to be prescribed in the Memphis–Shelby region (OR: 0.93, 95% CI: 0.87–0.99) and more likely to be prescribed in the Chattanooga-Hamilton (OR: 1.09, 95% CI: 1.00–1.18), Knox (OR: 1.10, 95% CI: 1.02–1.18), Northeast (OR: 1.13, 95% CI: 1.04–1.23), South Central (OR: 1.10, 95% CI: 1.01–1.20), and Southeast (OR: 1.18, 95% CI: 1.08–1.29) regions. (Fig 2 and Table 3)

## Discussion

Despite CDC and USPSTF guidelines recommending HCV screening for all Baby Boomers, in this study only 7% of those eligible for screening had documentation of an HCV antibody test. While this is likely an underestimate of the true number of insured Tennessean Baby Boomers screened for HCV, considering some individuals in the cohort may have received HCV antibody testing prior to 2013 or independent from their health insurance, this still highlights a significant service gap. Studies conducted in other parts of the US have reported HCV screening rates ranging 1% to 21% [9–13], emphasizing that TN is not alone in needing to improve implementation of HCV screening recommendations. However, considering that TN has among the highest rates of HCV in the country [2], there should be an increased sense of urgency for improving HCV screening rates.

Complete data on test results and treatment outcomes would be required to comprehensively assess linkages to subsequent steps along the chronic HCV CoC. Ideally, the proportion of those who received an HCV PCR test among those who had a positive HCV antibody test, and the proportion of those evaluated and treated for HCV among those who had a positive HCV PCR test would be reported. However, the data utilized for this study had very few HCV antibody and PCR test results available. So instead, proportions receiving various services along the chronic HCV CoC among those who received any HCV-related service were determined. This approach is limited such that true levels of attrition at each step of the diagnostic and treatment cascade cannot be determined from this data. That said, assuming a population prevalence at least equal to the national average of approximately 1% [1], and assuming that most of those who were evaluated for HCV treatment (n = 3,690; 0.7% of this cohort) and prescribed HCV medications (n = 1,731; 0.3% of this cohort) had true chronic HCV infections, then many chronically HCV-infected Tennessean Baby Boomers did not access and potentially benefit from HCV services. With the introduction of safe and effective anti-HCV DAAs, these results raise concerns about the health of Tennessean Baby Boomers and highlight opportunities for engaging them in the HCV CoC.

Understanding demographic differences in uptake along the HCV CoC also has the potential to inform public health interventions aimed at mitigating the individual and population level effects of chronic HCV. Non-Hispanic blacks are known to have the highest prevalence of HCV in the United States [14]; however, in this study non-Hispanic blacks were only slightly more likely than non-Hispanic whites to be screened and/or treated for HCV. This is consistent with findings from studies in Michigan and Pennsylvania [13, 15]. On the other hand, HCV rates for Hispanics increased by 13.6% from 2013 to 2014 [16], and in this study Hispanics were significantly more likely to be screened and/or treated for HCV than non-Hispanic whites. According to the National Health and Nutrition Survey, men are 1.7 times more likely to be infected with HCV than women [14]; however, in this study women were slightly more likely than men to be screened for HCV, and men were only slightly more likely than women to be treated for HCV. The reasons underlying these racial/ethnic and sex differences in linkage to HCV screening and treatment services are not entirely known, but may be a reflection of patient and provider perceptions of HCV risk. Perhaps targeted educational initiatives could enhance access to HCV screening and treatment for those groups at highest risk.

There were also regional differences in HCV screening and treatment. HCV screening was more likely to occur in the Nashville-Davidson region than in other regions of TN, and people from the Jackson-Madison and Sullivan regions were least likely to be screened for HCV. Those in the Memphis-Shelby region were least likely to be treated for HCV. Perhaps lessons learned from higher performing regions could be applied to the lower performing regions. Interestingly, the eastern regions of Tennessee, which seem to be most affected by recent increases in *acute* HCV incidence, are not necessarily the same regions underperforming in *chronic* HCV screening and treatment. Strategies to combat these related but distinct issues should be individualized and evidence based.

Strengths of this study include its large sample size and utilization of data from all regions of TN; this is the largest assessment of the chronic HCV CoC in TN to date. However, data were not collected or analyzed from uninsured individuals or those insured by other health insurers, and therefore these results may not be generalizable to all Tennessean Baby Boomers. While this is a major limitation, the collaborating health plan insures approximately 60% of Tennesseans, and those who are uninsured or under-insured would have very limited access, if any, to the HCV diagnostics and treatments that are the focus of this study. Another potential limitation was the excessive absence of race/ethnicity data; however, this limitation was minimized by utilizing TN census data to impute race/ethnicity proportionate to the known distribution in the various regions of the state. That said, results such as the strongly negative association between non-Hispanic multi-race and HCV screening must be interpreted with caution due to the high-level of imputation in a relatively small group. The lack of test results and treatment outcomes also limits this study’s ability to comprehensively assess linkages to services along the chronic HCV CoC. These limitations highlight the importance of building capacity for comprehensive HCV surveillance activities at the state level. It might also be mutually beneficial if insurers are better able to gather and share more detailed data about populations and access to health services with public health authorities.

## Conclusion

Many insured Tennessean Baby Boomers do not access HCV screening services, despite national recommendations. Demographic and regional differences in uptake along the chronic HCV CoC should inform public health interventions aimed at mitigating the individual and population level effects of chronic HCV. Improved strategies for collection and use of HCV health services and outcomes data are needed.
